# A survey for potentially zoonotic parasites in backyard pigs in the Bucaramanga metropolitan area, Northeast Colombia

**DOI:** 10.14202/vetworld.2021.372-379

**Published:** 2021-02-09

**Authors:** Juan Carlos Pinilla, Elsa Morales, Angel Alberto Florez Muñoz

**Affiliations:** 1 Department of Veterinary Medicine, Research Group in Agricultural Sciences, Faculty of Exact, Natural and Agricultural Sciences, University of Santander, Bucaramanga, Colombia; 2 Department of Microbiology, Research Group in Clinical Sciences (CliniUdes), Faculty of Health, University of Santander, Bucaramanga, Colombia

**Keywords:** Colombia, cysticercosis, parasite, pigs, *Trichinella*, zoonotic

## Abstract

**Background and Aim:**

Backyard pigs farming is a rearing system associated with poor hygienic and sanitary conditions of the pig, often causing public health and food safety problems. Therefore, this study was conducted to investigate the occurrence of potentially zoonotic parasites in population pig reared under backyard farming in the Bucaramanga metropolitan area, Northeast Colombia.

**Materials and Methods:**

From September to December 2019, a total of 558 fecal samples from 64 backyard pig farms were examined for the presence of enteric protozoan infection. The coprological diagnosis was done by direct examination using Lugol’s iodine solution, buffered saline solution, and Kinyoun technique. In addition, blood samples were collected from 200 pigs. Serum was collected and used for the detection of *Trichinella spiralis* and *Taenia solium* cysticercosis infections, using the enzyme-linked immunosorbent assay.

**Results:**

The overall prevalence of zoonotic protozoa in the Bucaramanga metropolitan area was 65.2%, reporting 52.7% prevalence for *Balantidium coli*, 33.7% for *Entamoeba coli*, and 5.7% for *Cryptosporidium* spp. Regarding the prevalence by municipalities, there was no statistical association (p>0.05), indicating that the prevalence was similar in the region under study. Pigs >7 months of age showed to be a risk factor for *B. coli* infection, indicating that the prevalence increases with the age, while pigs raised at >1000 masl and access to latrines, increased infection risk for *E. coli* and *Cryptosporidium* spp. infections. In the present study, *T. spiralis* infection was not detected in the analyzed sera, while *T. solium* cysticercosis infection was found to be 40.5%.

**Conclusion:**

The high prevalence of protozoan infections and porcine cysticercosis reported in this study could be due to poor facilities, and lack of hygiene in the facilities, and suggests the possible transmission of these parasite populations between pigs and humans, thus increasing the transmission of parasites zoonotic potential. Therefore, appropriate sanitary management practices and deworming programs should be adopted to reduce the prevalence of these infectious agents.

## Introduction

In Colombia, pig farming is a technified industry that supplies the national market; however, backyard pig farming systems have been an alternative to generate economic income in many families in different regions of the country [1]. In general, this type of rearing system is associated with low sociocultural status, poor facilities, absence of veterinarian, poor hygienic and sanitary conditions of the pig, lack of wastewater treatment, and the development of parasitic diseases, often causing public health and food safety problems, especially due to the transmission of zoonotic parasites [2].

Many parasites affect swine health and can be transmitted to humans. As for protozoan, *Balantidium coli*, *Entamoeba coli*, and *Cryptosporidium* spp. are the main species [3], while *Taenia solium* and *Trichinella spiralis* represent the species of helminths with the greatest impact on public health worldwide [4]. In the Cundinamarca department, Colombia, Pulido-Villamarín *et al*. [5], Mendoza-Gómez *et al*. [6] reported a high frequency of *E. coli* and *B. coli* cysts in semi-technical pig farms, while *E. coli* cyst and *T. solium* eggs were the most prevalent zoonotic parasites found in fecal samples from backyard pigs. Furthermore, Cazorla *et al*. [7] and de Guzman *et al*. [8] registered prevalence of 45.4% and 65% for *B. coli* infection, respectively, in pigs from rural communities of Venezuela. On the other hand, Agudelo-Florez and Palacio [9] reported a seroprevalence of *T. solium* antibodies between 2.33% and 6.84% in pigs from endemic areas of Colombia, while [10] overall seroprevalence of human cysticercosis in Colombia is reported 8.55%. However, *Trichinella spiralis* has not yet been reported in Colombia [11,12].

In Colombia, there is little epidemiological information on potentially zoonotic parasites in pigs, especially in the Northeastern regions. The aim of the present research was to investigate the occurrence of potentially zoonotic protozoa, as well as, the seroprevalence of *T. solium* porcine cysticercosis and *T. spiralis* in pigs reared under backyard farming in the Bucaramanga metropolitan area, Northeast Colombia.

## Materials and Methods

### Ethical approval

This research was approved by the Institutional Ethical Committee of the University of Santander and Industrial University of Santander (ref No CIF0311-19).

### Study area and period

The Santander department is located in the Colombian Northeast. This region is characterized by agricultural and cattle livestock production. According to the Colombian Agricultural Institute (ICA), the swine population census for the department was 93,000 pigs, being 85% in backyard and 40% located in the Bucaramanga metropolitan area [13]. This region comprises a geographical area of 1479 km^2^. Rainfall is regular throughout the year; however, more rains are experienced in October-December. Bioclimatic characteristics of the region are very similar and with a mean annual temperature of 25°C, with little weather variation throughout the year. The altitude is between 600 and 1700 masl and the mean annual rainfall is 1040 mm, with 78% relative humidity [14]. In general, the meat pork produced in this region is offered to local markets for internal consumption. This study was conducted from September to December 2019.

### Sampling design

**Figure-1:**
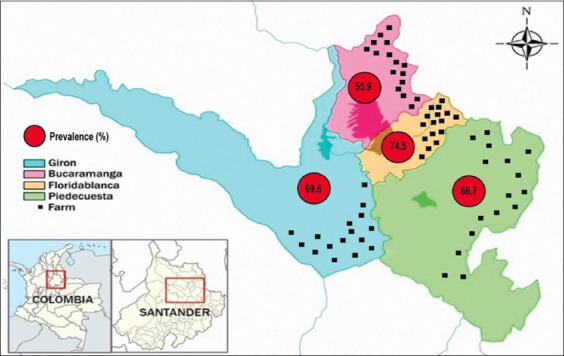
Bucaramanga metropolitan area map showing the sampling sites (black dots) and the overall prevalence of zoonotic protozoa [Source: https://es.wikipedia.org/wiki/%C3%81rea_metropolitana_de_Bucaramanga].

A cross-sectional and descriptive study was designed. Based on the ICA’s inventory records, the farms were visited and selected those whose owners agreed to participate in the study. The geographical locations of the sampling site are shown in Figure-1. Sixty-four backyard pig farms located in the Bucaramanga metropolitan area, were visited from September to December 2019 (wet period). Most of the pigs sampled were crossbreeds between the Yorkshire, Landrace, and Pietrain breeds. For zoonotic protozoa, the sample size was calculated to estimate the municipality level prevalence and considering an infinite population using the formula provided by Thrusfield [15]: n=[Zα^2^ P (1-P]/d^2^, where “n” is the required sample size; “Z” is the multiplier from a standard normal distribution (α=1.96) at a probability level of 0.05; “p” is the expected prevalence which is most conservatively estimated to be 50 %, considering that there are no reference data from pigs in the area under study; (1−P) is the probability of having no disease; and “d” is the maximum associated error (5%). Therefore, a sample size of 384 pigs was determined for the study. However, to increase precision, a sample size of 558 pigs was planned. In addition, the sample size for the serological study was calculated using the same formula and with a reported prevalence by Flórez *et al*. [10] of 8.5% for human cysticercosis in the country, an “n” of 200 animals was determined. Animals were categorized according to the age in: ≤2 months, 3-6 months, 7-12 months, and ≥13 months [16]. Epidemiological variables were obtained using a questionnaire administered to the owner or manager of each herd at the time of sampling. These data included sex, that is, male and females, pigs free ranging, location of the farm, pig access to latrines, dewormed, type of feed supplied to the pigs, and water source for the animals. During the sampling, no clinical signs of parasitic infection were observed in examined pigs.

### Sample collection and laboratory analysis

A total of 558 fecal samples were collected from backyard pigs. Approximately 5 g of feces were collected directly from the rectum from each pig, using previously labeled polyethylene bags. In suckling piglets, a swab was introduced rectally to collect a small fecal sample. Samples were placed into containers filled with ice packs and immediately transported for processing in the Veterinary Clinic Research Laboratory of the University of Santander. Some of the fecal samples were immediately processed for direct examination with Lugol’s iodine solution (1:5 dilutions) and buffered saline solution to determine *B. coli* and *E. coli* cysts [17]. Another portion of the fecal sample was processed by the Ziehl–Neelsen technique to detect *Cryptosporidium* spp. oocysts [17]. The parasitic species observed were identified by the morphology of their oocysts and cysts using a light optical microscope with 40× and 100× [17]. For serum, 5 mL whole blood was collected by jugular venipuncture in a sterile Vacutainer tube without ethylenediaminetetraacetic acid. The serum was separated and clarified by centrifugation at 3000 rpm for 10 min and stored at −20°C till the tests were performed. Serum samples were analyzed by indirect enzyme-linked immunosorbent assays (ELISA), according to the manufacturer’s instructions. These tests were done using an indirect ELISA kit for the detection of anti-*T. spiralis* antibodies (ID Screen®, Grabels, France) and Monoscreen ELISA kit for antigenic diagnosis of *T. solium* cysticercosis (Bio-X Diagnostics, Rochefort, Belgium).

### Statistical analysis

The results were analyzed by the Chi-square test to evaluate the association between variables (municipality, sex, age group, altitude, free-ranging pigs, access to latrines, deworming, type of food, and water source) and prevalence values. Odds ratio (OR) and their confidence intervals for risk factors were obtained by univariable logistic regression analysis, taking as the reference category those with the lowest probability of risk, and leaving the others as study categories [18]. The level of significance for the analyses was 5%. Calculations were made using the SPSS® Statistics for Windows, (IBM, USA) version 21.0 [19].

## Results

### Prevalence of zoonotic protozoa

The overall prevalence of zoonotic protozoa in the Bucaramanga metropolitan area was 65.2% (364/558). There was no statistical association (p>0.05) between prevalence values in the four municipalities: 55.9% (104/186) in Bucaramanga, 74.5% (70/94) in Floridablanca, 69.6% (110/158) in Giron, and 66.7% (80/120) in Piedecuesta (Figure-1). In most (60.1%) of the positive fecal samples, monoparasitism was evidenced, while in 39.9% of the cases, two or three protozoan parasites were found. In these cases of polyparasitism, 92.1% showed coinfection between two protozoa, being the coinfection between *B. coli*×*E. coli* the most frequent. Overall infection rate was 52.7% for *B. coli*, 33.7% for *E. coli*, and 5.7% for *Cryptosporidium* spp. No statistical association was found (p>0.05) between *B. coli* and *Cryptosporidium* spp. infections and the municipalities indicating that the prevalence is present in similar proportions in the four municipalities (Table-1).

**Table-1:** Prevalence of zoonotic protozoan obtained from the fecal samples of backyard pigs in the Bucaramanga metropolitan area, Santander, Colombia.

Protozoan	Municipality				Overall	Chi-square test	
	
	Bucaramanga (n=186)	Floridablanca (n=94)	Piedecuesta (n=120)	Giron (n=158)		χ^2^-value p-value	
				
	Positive (%)	Positive (%)	Positive (%)	Positive (%)	Positive (%)		
*Balantidium coli*	88 (47.3)	54 (57.4)	62 (51.7)	90 (57)	294 (52.7)	2.1	0.55
*Entamoeba coli*	36 (19.4)	46 (48.9)	44 (36.7)	62 (39.2)	188 (33.7)	14.7	0.002
*Cryptosporidium* spp.	12 (6.5)	8 (8.5)	4 (3.3)	8 (5.1)	32 (5.7)	1.46	0.69

Statistically significant (p<0.05)

### Risk factors for protozoal infections

There was a statistical association (p<0.05) between the positive values of *B. coli* with respect to age group. In this sense, 7-12 months and ≥13 months groups showed 1.8 (OR=1.8; p<0.05) and 2.9 (OR=2.9; p<0.05) times probability for infection than other age groups. Infection rate increases with the age of the animal. The rest of the variables analyzed showed not to be a risk factor for this parasite (Table-2). In relation to *Cryptosporidium* spp., pigs with access to latrines showed almost 5 times higher probability for infection (OR=4.5; p<0.05) than those with no access to latrines. The rest of the variables were not shown to be a risk factor for this protozoan (p>0.05) (Table-3). On the other hand, pigs raised >1000 masl, showed a higher probability of *E. coli* infection (OR=1.7; p<0.05), than those kept on farms <1000 masl. The rest of the variables were not shown to be a risk factor for this protozoan (p>0.05) (Table-4).

**Table-2:** Results of logistic regression analysis for *Balantidium coli*: Risk factors.

Variables	*Balantidium coli*			
	
Age group	n	%	p-value	OR
≤2 months	142	28.1		1
3-6 months	98	42.8		0.94
7-12 months	176	62.5		1.8
≥13 months	142	71.8	0.01	2.9
Sex				
Male	214	56.1		1
Female	344	50.6	0.37	0.8
Altitude				
<1000	278	48.9		1
>1000	280	56.4	0.21	1.3
Free-ranging pigs				
No	426	52.6		1
Yes	132	53	0.95	0.9
Access to latrines				
No	514	51.8		1
Yes	44	63.6	0.28	1.6
Deworming				
No	34	52.9		1
Yes	524	52.7	0.9	0.9
Type of food				
Restaurant residues	120	56.7		1
Concentrate	112	46.4		0.66
Mixed	326	53.4	0.5	0.87
Water source				
Spring water	130	46.2		1
Deep well water	428	54.7	0.2	1.44
Overall	558	52.7		

Statistically significant (p<0.05). OR=Odds ratio, 1=Reference category

**Table-3:** Results of logistic regression analysis for *Cryptosporidium* spp.: Risk factors.

Variables	*Cryptosporidium* spp.			
	
Age group	n	%	p-value	OR
≤2 months	142	2.8		1
3-6 months	98	2.0		0.7
7-12 months	176	10.2		3.9
≥13 months	142	5.6	0.13	2
Sex				
Male	214	6.5		1
Female	344	5.2	0.65	0.7
Altitude				
<1000	278	5		1
>1000	280	6.4	0.62	1.2
Free-ranging pigs				
No	426	5.6		1
Yes	132	6.1	0.89	1.08
Access to latrines				
No	514	4.7		1
Yes	44	18.2	0.009	4.5
Deworming				
No	34	5.9		1
Yes	524	5.7	0.9	0.9
Type of food				
Restaurant residues	120	3.3		1
Concentrate	112	3.6		1.07
Mixed	326	7.4	0.38	2.3
Water source				
Spring water	130	3.1		1
Deep well water	428	6.5	0.3	2.2
Overall	558	5.7		

Statistically significant (p<0.05). OR=Odds ratio, 1=Reference category

**Table-4:** Results of logistic regression analysis for *Entamoeba coli*: Risk factors.

Variables	*Entamoeba coli*			

Age group	n	%	p-value	OR
≤2 months	142	32.4		1
3-6 months	98	28.6		1
7-12 months	176	35.2		1.2
≥13 months	142	36.6	0.8	1.2
Sex				
Male	214	33.6		1
Female	344	33,7	0.9	1
Altitude				
<1000	278	28.1	1
>1000	280	39.3	0.04	1.7
Free-ranging pigs				
No	426	32.4		1
Yes	132	37.9	0.41	1.2
Access to latrines				
No	514	34.2		1
Yes	44	27.3	0.5	0.7
Deworming				
No	34	35.3		1
Yes	524	33.6	0.88	0.99
Type of food				
Restaurant residues	120	40		1
Concentrate	112	21.4		0.41
Mixed	326	35.6	0.08	0.86
Water source				
Spring water	130	33.8		1
Deep well water	428	33.6	0.9	0.9
Overall	558	33.7		

Statistically significant (p<0.05). OR=Odds ratio, 1=Reference category

### *T. spiralis* and *T. solium* cysticercosis infections

In general, 200 samples of swine sera were examined by ELISA, and none showed anti-*T. spiralis* antibodies. In contrast, the seroprevalence of porcine cysticercosis was 40.5% (81/200). These values were analyzed by univariable logistic regression, and according to the results obtained, there was a statistical association (p<0.05) between the seropositive values for porcine cysticercosis with respect to the altitude, free-ranging pigs, access to latrines, and deworming. Therefore, free pigs and farms at >1000 masl showed 2 (OR= 2; p<0.05) and 2.9 (OR= 2.9; p<0.05) times, respectively, the higher probability of infection than non-free pigs confined to farms at <1000 masl. While access to latrines and non-dewormed pigs showed 2 (OR=2; p<0.05) and almost 3 (OR=2.7; p<0.05) times higher risk of infection of *T. solium*. There was no statistical significance between the porcine cysticercosis concerning to the other variables analyzed (p>0.05) (Table-5).

**Table-5:** Seroprevalence of porcine cysticercosis *T. solium* antigen in backyard pigs from the Bucaramanga metropolitan area.

Variable	n	*T. solium antigen*		

		%	p-value	OR
Municipality				
Bucaramanga	91	33		1
Floridablanca	29	51.7		1.4
Piedecuesta	45	37.8		0.6
Giron	35	54.3	0.087	2.04
Age group				
≤2 months	46	41.3		1
3-6 months	61	32.8		0.6
7-12 months	50	48		1.4
≥13 months	43	41.9	0.43	0.9
Sex				
Male	73	49.3		1
Female	127	35.4	0.054	0.56
Altitude				
<1000	121	33.9		1
>1000	79	50.6	0.02	2
Free-ranging pigs				
No	41	22		1
Yes	159	45.3	0.007	2.9
Access to latrines				
No	69	27.5		1
Yes	131	47.3	0.03	2
Deworming				
Yes	8	0		1
No	192	42.2	0.02	2.7
Type of food				
Restaurant residues	32	46.9		1
Concentrate	48	50		1.9
Mixed	120	35	0.14	0.6
Water source				
Spring water	30	36.7		1
Deep well water	170	41.2	0.64	1.2
Overall	200	40.5		

Statistically significant (p<0.05). OR=Odds ratio, 1=Reference category, *T. solium*=*Taenia solium*

## Discussion

Many of the pathogens that affect pig production can be transmitted to humans. Among the porcine, parasites widely recognized as zoonotic are *B. coli*, *Toxoplasma gondii*, *Cryptosporidium* spp., *T. solium* and its metacestode, and *T. spiralis*. Therefore, the importance of these zoonoses at a global and regional level lies directly in human and animal health and indirectly in the socioeconomic development of many peoples [5]. Despite the importance of porcine zoonoses, few studies have been done in Colombia. In Latin America, the prevalence of *B. coli* infection in humans ranges between 0.5% and 2.1%, being low compared to other intestinal infections by protozoa. However, Mendoza-Gómez *et al*. [6] revealed 10% infection for *B. coli* and 60% for *E. coli* in workers from semi-technified farms located in the department of Cundinamarca, Colombia. On the other hand, Flórez *et al*. [10] determined a seroprevalence of 1.96% of human cysticercosis in the department of Santander, Colombia.

### Prevalence of zoonotic protozoa

The overall prevalence of zoonotic protozoa found in the four municipalities was similar, since the temperature and humidity conditions in the zone, the management in most of the farms, as well as the programs in the control of infectious agents are similar in the four municipalities [20]. Furthermore, the high prevalence found in this study indicates the absence of hygienic and sanitary conditions in the examined farms, where some risk factors may have favored the dissemination and transmission of protozoan parasites among animals. The results obtained in this research agree with those reported by several authors [7,16,21,22] who found similar values of prevalence in backyard pig farming from Colombia, Venezuela, Mexico, and Brazil.

The present study found that *B. coli* was the most prevalent zoonotic protozoan (52.7%) followed by *E. coli* (33.7%). These results obtained agree with those recorded by de Guzman *et al*. [8] and Pinto *et al*. [23] who revealed similar values of prevalence in outdoor pigs. However, the results obtained differ from those reported by Mendoza-Gómez *et al*. [6] who found 5% prevalence for *B. coli* in farms from Cundinamarca department, Colombia. The prevalence of *E. coli* in the present study was 33.7%, and this result agrees with those reported by Mendoza-Gómez *et al*. [6] who found 40% prevalence in pigs from Cundinamarca department. Although *E. coli* do not cause infection, their presence indicates the fecal-oral transmission in the host, which is an indicator for the general assessment of the hygiene status of the animals. According to the results obtained, the *E. coli* prevalence was higher (48.9%) in pigs from Floridablanca municipality. This region is located at medium altitudes (1000-1700 masl), with mean annual temperature (25-30°C) long the year, with prolonged periods of rain and average precipitation rates between 1100 and 1400 mm, which are favorable climatological conditions for this protozoan and increased risk of infection for the animals. Moreover, this result could be caused by the ingestion of contaminated water or food or by immunosuppression in the animals due to factors like stress associated with overcrowding.

About *Cryptosporidium* spp., the prevalence rate found was 5.7%. This result agrees with those by Mendoza-Gómez *et al*. [6] who found similar prevalence values in semi-technified farms. Cryptosporidiosis is a disease of high zoonotic importance, known as a public health problem that affects mainly people that interact with farm animals daily. Therefore, it is important to demonstrate the presence of this pathogen with zoonotic potential, to take measures such as strengthening the breeding management of pigs and improving the sanitary control to avoid the spread of pathogens [24].

### Risk factors for protozoan infections

The access to latrines in some of the examined farms showed to be a risk factor for *Cryptosporidium* spp. infection. These results agree with those reported by Thomas *et al*. [25] who found a positive correlation between parasitic infection and interactions with latrines, and a moderate positive correlation between coccidia infection and home range area [25]. These results could be explained since this pathogen is transmitted through the fecal-oral route in humans and animals, usually through the ingestion of contaminated water or food with feces [26]. Probably, the contact with infective human fecal material by pigs is an important requisite for the successful maintenance of the parasite lifecycle; therefore, it would be to think stands to reason that keeping free-ranging pigs in contact with latrines, would increase the risk of the pigs in acquiring this parasitic infection [25]. In developing countries, the environmental risk factors and routes of transmission for *Cryptosporidium* spp. infection are not well defined. Despite the numerous surveillance studies reported, few investigations have been conducted on the source of infection. However, contamination of water supplies and infection of domestic animals, lacking adequate municipal water and sewage services, and using a field or latrine for defecation in human communities were correlated with a higher risk of cryptosporidiosis [26]. Regarding the age factor, pigs >7 months of age showed higher prevalence values for *B. coli* than those younger (<7 months). This could be explained because young pigs, especially suckling piglets and weaner, are usually less affected by this parasitic infection, but soon became infected either from the mother or from coprophagy of residual fecal material. In general, however, the prevalence increases in pigs with age [27]. Altitude factor showed to be a risk factor for *E. coli*, since all the backyard farms in the Floridablanca municipality are > 1000 masl.

### *T. spiralis* and *T. solium* cysticercosis infections

In the present study, no *T. spiralis* antibodies were detected in the porcine sera analyzed, which could indicate that this parasite does not circulate in the backyard pigs population of the region under study. These results agree with those reported by Chaparro-Gutiérrez *et al*. [28] and Pulido-Villamarín *et al*. [29] who did not report the infection by this nematode in pigs from different regions of Colombia. The results obtained in the present study disagree with those reported by several authors [30,31], where this zoonotic pathogen is endemic in Argentina and Chile, and it’s circulating among a large number of animals including pigs. These results also differ from those found by Pozio [32] who reported that anti-*Trichinella* antibodies were detected in sera from domestic pigs in different regions of Bolivia. Despite serology can occasionally yield false-positive results Chávez-Larrea *et al*. [33], it would be warranted that any seropositive result may be confirmed by the artificial digestion assay [28]. A study showed that when the recommended protocol for the ELISA test is strictly followed, a negative result is an excellent indicator of the absence of infection, with a specificity of 98.29% [34]. Despite our results, it would be important to maintain a continuous vigilance of the parasite in our country, considering that the endemic situation in Argentina, Chile, and Bolivia, in addition to the effects of globalization, could be possible mechanisms for the spread of the parasite for our backyard pigs and other animals. In this sense, serological tests for the diagnosis of this parasite have the advantage of detecting mild infections (<1 larva per gram of muscle) and they can perform on the living animal. However, these methods do not replace direct detection methods that are carried out in slaughterhouses to control this zoonosis due to its high costs, but they are suitable for control programs on farms as well as for epidemiological studies of the cycle jungle disease [11].

The seroprevalence of *T. solium* cysticercosis was 40.5% which is similar to the seroprevalence of porcine cysticercosis found in areas where the disease is considered hyperendemic [35]. Few studies reveal the presence of porcine cysticercosis in Colombia; however, our results agree with those reported by Molano *et al*. [36] who demonstrated a similar serological prevalence in semi-technical and backyard farms in the Boyaca department, Colombia. Our study revealed that the altitude, no deworming, and free-ranging pigs increased the risk of being seropositive to porcine cysticercosis *T. solium*. In this sense, the coexistence of poor sanitary conditions and free-ranging pigs certainly plays an important role in the circulation of *T. solium* infection in this region. Free-roaming of pigs is known to be an important risk factor for *T. solium* infection of pigs [37]. Probably, pigs free-ranging when they have access to latrines, could have higher contact with *T. solium* eggs, and therefore, increase the odds of cysticercosis transmission risk to the pig itself, to other pigs, even humans [25]. However, other studies consider that the presence of latrines is a protective factor in decreasing the seroprevalence of porcine cysticercosis and is not surprising as the use of latrines has been proposed to control cysticercosis worldwide by several authors [37,38].

The pig, as an intermediate host, plays an important role in *T. solium* taeniasis, due to its coprophagic habits as it sometimes has access to latrines, where it can feed on excrement of an infected host (man), so backyard pigs are considered of high risk, especially if not kept in proper hygiene and feeding conditions and if their meat is consumed without proper cooking [39]. Therefore, the results obtained allow us to affirm that porcine cysticercosis represents a risk for public health in the region under study, since socioeconomic and cultural aspects are involved in the life cycle of this parasite, with human activities being those directly involved in reproduction and dispersal of this parasite [40].

## Conclusion

It concluded that the high prevalence of protozoan infections and porcine cysticercosis reported in this study could be due to poor facilities, and lack of hygiene in the facilities, and suggests the possible transmission of these parasite populations between pigs and humans, thus increasing the transmission of potentially zoonotic parasites. Therefore, appropriate sanitary management practices and deworming programs should be adopted to reduce the prevalence of these infectious agents.

## Authors’ Contributions

JCP conceived and designed the research. JCP and AAF conducted the sample collection. JCP and EM processed samples in the Microbiology Research Laboratory. JCP carried out the data analysis and writing of the manuscript. AAF edited the manuscript. All the authors read and approved the submitted version of the manuscript.
